# Association of Hepatitis C Virus With Insulin Resistance: Evidences From Animal Studies and Clinical Studies

**Published:** 2012-01-20

**Authors:** Sadaf Badar, Bushra Khubaib, Muhammad Idrees, Abrar Hussain, Zunaira Awan, Sadia Butt, Samia Afzal, Madeeha Akram, Zareen Fatima, Mahwish Aftab, Sana Saleem, Sara Munir, Bisma Rauff, Mahrukh Naudhani, Liaquat Ali, Muhammaad Ali, Irshadul Rehman

**Affiliations:** 1 National Centre of Excellence in Molecular Biology, University of the Punjab, Lahore, Pakistan

**Keywords:** Insulin Resistance, NS5a Protein, Hepatitis C

## Abstract

**Context:**

HCV infection is strongly associated with development of insulin resistance and type-2 diabetes, however molecular mechanism of these associations is not known. The aim of this review was to conduct a comprehensive literature search to understand the nature of the association between hepatitis C virus (HCV) infection and insulin resistance (IR). We also explored the role of HCV core protein and NS5a in modulating the course of the insulin-signaling pathway.

**Evidence Acquisitions:**

We searched Directory of Open Access Journals (DOAJ) Google Scholar, Pubmed (NLM), LISTA (EBSCO), Web of Science (TS and PakMediNet).

**Results:**

Emerging evidence suggests an association between HCV infection and carotid/coronary vascular disease. IR appears to be a dominant underlying cause of accelerated atherosclerosis in patients with chronic hepatitis C (CHC). HCV can induce IR directly through the stimulation of SOCS3 and PPA2, and both of these molecules have been shown to inhibit interferon-α signaling. Improvement of insulin sensitivity may increase the response rate to antiviral treatment and prevent IR complications, including vascular diseases. The results of several clinical trials that have used insulin sensitizers (metformin and PPAR-γ agonists) have been inconclusive.

**Conclusions:**

Beside the association between HCV and IR, the published data also have showed the possible association of HCV core and NS5A protein with IR.

## 1. Context

Hepatitis C virus (HCV) infection is a serious health problem affecting over 130 million people worldwide [1]. Approximately 85% of the HCV-infected population develops severe complications, such as chronic HCV (CHCV), cirrhosis, diabetes, and hepatocellular carcinomas [2][3][4][5][6][7]. CHCV is reported to be the major cause of 50% to 76% of all liver cancers globally and also the reason for two-third of all cases of liver transplantation in developed countries [8]. It is recognized as a metabolic disease mainly because of its major role in the development of insulin resistance (IR) and type 2 diabetes mellitus (T2DM). The precise nature of association of IR and CHCV is not completely understood yet; however, it is an established fact that HCV infection enhances IR, which in turn causes liver failure and the development of hepatocellular carcinomas (HCCs) [8] by affecting the response rate for antiviral therapy [9]. Furthermore, old age, obesity, severe liver fibrosis, and family history of diabetes are the key risk factors for diabetes mellitus (DM). In non-diabetic HCV-infected patients, IR is associated with stages of liver fibrosis [10]. Several HCV-induced pathways lead to fibrosis, inflammation, steatosis, apoptosis, hepatocellular carcinoma, and insulin resistance [11][12][13][14]. An insight into the mechanisms of liver failure, fibrosis, steatosis, and oxidative stress will help understand the association between HCV pathogenesis and IR [15]. This review explores the role of CHCV in the development of IR and also analyzes the possible association of HCV core protein and NS5A protein with IR.

## 2. Evidence Acquisition

Directory of Open Access Journals (DOAJ) Google Scholar, Pubmed (NLM), LISTA (EBSCO), Web of Science (TS and PakMediNet) were searched with key words "Role of HCV in insulin resistance" " Key observations relevant to insulin resistance" , involvement of HCV in the development of insulin resistance" in recent 10 years. Thanks to Higher Education Commission (HEC) Government of Pakistan that we were able to obtain full articles on the reference lists from retrieved documents free of charges. As the information about this topic was rare, small clinical trials and case reports were also included.

## 3. Results

We were able to found 70 research and review articles relevant to this topic directly or indirectly. From the information given in these papers, we drew out following aspects.

### 3.1. Insulin

Insulin, which is synthesized by the pancreatic β cells, is the most effective anabolic hormone. It is important for cell growth, cell differentiation, protein synthesis, and maintenance of glucose levels in the body [8]. It regulates blood glucose levels by inhibiting the production of liver glucose and by promoting the absorption of glucose in muscle and adipose tissue [16]. Insulin stimulates glucose transporter type 4 (GLUT4), which translocates glucose into the plasma membrane, thus increasing cell glucose uptake [8]. It is also involved in lipid metabolism, suppressing lipolysis, and promoting lipid synthesis in liver, muscle, and adipose tissues [16]. Insulin resistance develops when the concentration of insulin in blood is insufficient to efficiently regulate glucose uptake [17].

### 3.2. Insulin Signal Transduction

Insulin-signaling pathway begins when insulin binds to the extracellular α subunit of insulin receptor substrate (IRS), which further phosphorylates the intracellular tyrosine-kinase domain of the β subunit of the insulin receptor. This phosphorylation produces docking sites for many other proteins having SH2 (Src homology 2) domains like phosphatidylinositol 3-kinase (PI3k) [18][19]. PI3k binds to IRS, which phosphorylates phosphatidylinositol 4,5-bisphosphate (PIP2) to phosphatidylinositol triphosphate (PIP3). Interaction of PIP3 with phosphosinositide-dependent kinase 1 (PDK1), in turn, activates protein kinase B (AKT/PKB) and protein kinase C (PKC) [20]. Once activated, AKT/PKB decreases the blood glucose levels mainly through 2 pathways. Firstly, by stimulating the phosphorylation of glycogen synthase, which converts surplus glucose into glycogen. Secondly, by suppressing glucose-6-phosphatase and phosphoenolpyruvate carboxykinase, which are involved in gluconeogenesis. Moreover, AKT is important for the localization of GLUT4 in the cell membrane and thus stimulates glucose uptake into skeletal muscle and adipose tissue [8]. Insulin resistance arises from the impairment of the above-stated insulin-signaling pathway at multiple steps. One of the most important steps is the phosphorylation of key serine residues including Ser307, Ser318, Ser636, and Ser639 and not phosphorylation of the tyrosine residues for the inhibition of IRS [8].

### 3.3. HCV Core Protein and IR

HCV core protein has been reported to induce overexpression of tumor necrosis factor-α (TNF-α) in the liver of transgenic mice and human hepatoma cell lines [21]. TNF is a proinflammatory cytokine that links obesity with IR [22]. Over expression of TNF causes insulin resistance through the inhibition of PI3K-mediated activation of insulin receptor substrate 1 (IRS-1) and insulin receptor substrate 2 (IRS-2), and tyrosine phosphorylation of the mitogen-activated protein kinases p42MAPK and p44MAPK. This phosphorylation leads to the impairment of glucose uptake in skeletal muscles [23] and also causes insulin resistance by inhibiting the IKK-β/NF-κB pathway [24]. Expression of core protein in hepatocytes causes an increase in the phospholrylation of ISR-1 at Ser312, impairs the activation of AKT at the Thr308 phosphorylation site and also impairs insulin-stimulated glucose uptake [25]. HCV core protein is also involved in the inhibition of tyrosine phosphorylation of IRS-1 and production of IRS-2 through a PA28γ-dependent pathway [21] However, it appears implausible that the HCV core protein is localized and degraded in the nucleus through a PA28γ-dependent pathway as a major mechanism for insulin resistance. HCV core protein may play an important role in the regulation of cellular inflammatory and immune responses through the activation of nuclear factor κ-light-chain enhancer of activated B cells (NF-κB) [26][27]. Activation of NF- κB is involved in the initiation of downstream production of cytokine (interleukin-6 [IL-6]) that leads to insulin resistance [28][29]. Increased production of TNF-α and suppressor of cytokine signaling-3 (SOCS-3) in HCV infection promotes IR mainly through the inhibition of the insulin receptor and tyrosine phosphorylation of IRS-1 [30]. The core protein causes improved production of SOCS-3, which leads to the degradation of IRS-1 and IRS-2 [31]. Although the effect of different genotypes of HCV on the insulin-signaling pathway is not yet clear, a significant clinical association has been found between the genotypes and IR [8]. Over expression of the core protein of genotype 3a and 1b in Huh7 cell line has no effect on the levels of IRS-2, but it reduces IRS-1 protein levels [32]. In the case of the core protein of genotype 3a, degradation of IRS-1 is mediated by downregulation of the production of peroxisome proliferator-activated receptor γ (PPARγ) and upregulation of SOCS-7 production; in contrast, genotype 1b core protein activates the mammalian target of rapamycin (mTOR) [32].

### 3.4. HCV NS5A and IR

The nonstructural protein 5A (NS5A) of HCV plays a significant role in virus-driven IR. HCV NS5A and increased endoplasmic reticulum stress in HCV infection contributes to overexpression of protein phosphatase 2A (PP2A) [33][34]. Christein and colleagues reported that PP2A modulates insulin-signaling pathway by dephosphorylation of AKT, leading to IR in CHC patients [35]. In their study, increased levels of PP2A were observed in liver biopsies of HCV patients and also in HCV-protein-expressing livers of transgenic mice. In both the models, insulin signaling was inhibited through PP2A-catalyzed dephosphorylation of AKT [35]. Moreover, PP2A can also impair interferon signaling, which is responsible for poor response to interferon therapy for IR in HCV patients [36].

### 3.5. Major Disorders Associated With HCV-Induced Insulin Resistance

IR is mainly categorized into 2 types-HCV-induced IR and lifestyle-associated IR. Pathogenesis of HCV-induced insulin resistance is different from that of lifestyle-associated IR [37]. Cardiovascular diseases are the main cause of death of patients with lifestyle-associated IR [37][38], while HCV-induced IR is responsible for many HCV-associated disorders [37] such as hepatic fibrosis, antiviral drug resistance, steatosis, and hepatocarcinogenesis.

### 3.6. Hepatic Fibrosis

IR plays a pivotal role in accelerating the severity of hepatic fibrosis in HCV-infected patients [31][37][39][40], which is the main characteristic of the early stages of CHCV infection [10]. It is because, IR stimulates the production of free fatty acids and increases lipid deposition in the liver, which, in turn, enhances oxidative stress and consequently results in the development of liver fibrosis [41][42][43]. Moreover, IR is also involved in the proliferation of hepatic stellate cells, which are the main site of deposition of abnormal extracellular matrix in hepatic fibrosis [44][45].

### 3.7. Hepatic Steatosis

Liver steatosis is frequently observed in patients with CHCV infection [46]. The rate of incidence of hepatic steatosis in HCV-infected patients is approximately twice than the prevalence rate of steatosis in people with other common liver disorders such as hepatitis B [47][48]. One of the major factors responsible for the development of steatosis in HCV-infected patients is the production of triglycerides [49]. The core protein of HCV induces aggregation of triglycerides (a low-density lipoprotein) through many mechanisms like activation of fatty acid synthase, downregulation of microsomal triglyceride transport protein, reduction of PPARγ, and upregulation of sterol regulatory element-binding protein-1c [49][50][51]. HCV core protein is also responsible for the production of reactive oxygen species, which, in turn, trigger lipid peroxidation, thus resulting in severe damage to the liver and ultimately to the development of steatosis [49][52]. These findings show a tight association of HCV with hepatic steatosis. Moreover, hepatic insulin resistance mediates the failure of the insulin-signaling pathway, leading to molecular and cellular changes that result in excess accumulation of triglycerides in the hepatocytes [53][54][55].

### 3.8. Hepatocarcinogenesis

A number of risk factors, such as male sex, age, cirrhotic liver, and alcohol consumption are involved in the development of hepatocellular carcinoma (HCC) in HCV-infected patients [56][57][58][59]. Recent studies show that IR is another key factor that plays a crucial role in the progression of liver cancer in HCV-infected patients [1][60][61]; however, the precise nature of the association is unknown. IR is responsible for the excessive production of free fatty acids [62][63], which causes increased liver adiposity, thus resulting in a reduction in the adiponectin levels and consequently the development of HCC [64]. In addition, fatty acid deposition in hepatocytes causes oxidative stress, which is another risk factor responsible for hepatocarcinogenesis [60][61].

### 3.9. Antiviral-Drug Resistance

Resistance to antiviral treatment is another serious problem associated with HCV-induced IR [37][65][66][67]. Although, the exact relationship between IR and poor response to antiviral treatment is not clearly understood, IR is known to cause an accumulation of hepatic lipids important for HCV replication [62][63]. These droplets activate viral replication and eventually lead to resistance to antiviral treatment not only in HCV genotype 1 but also in genotypes 2 and 3 [68]. Moreover, SOCS-3 production is upregulated by the HCV core protein, which is involved in reducing interferon activity. [69][70].

## 4. Conclusions

In this review, we have summarized the association between HCV and IR and also have analyzed the possible association of HCV core and NS5A protein with IR. IR is mainly categorized into 2 types-HCV-induced IR and lifestyle-associated IR. The pathogenesis of HCV-induced IR is different from that of lifestyle-associated IR. Cardiovascular diseases are the main cause of death in the case of lifestyle-associated IR, and HCV-associated IR is responsible for many HCV-related conditions such as hepatic fibrosis, antiviral-drug resistance, steatosis, and hepatocarcinogenesis. IR arises from the impairment of the insulin-signaling pathway at multiple steps. Various studies have reported that the core protein of HCV induces IR mainly by modulating the insulin-signaling pathway at the level of IRS. In the case of core proteins of genotype 3a, degradation of IRS-1 is mediated by the downregulation of PPARγ production and upregulation of SOCS-7 production; in contrast genotype 1b's core protein activates mTOR. HCV NS5A and increased endoplasmic reticulum stress in HCV infection contributes to the overexpression of PP2A, which leads to the impairment of interferon signaling, and is thus responsible for poor response to interferon therapy for treating IR in HCV-infected patients. Since the exact mechanisms of the molecular pathways of HCV-induced IR have not yet been understood, further research is required to determine how virus-induced IR can be managed.
